# Snapshots of Catalysis in the E1 Subunit of the Pyruvate Dehydrogenase Multienzyme Complex

**DOI:** 10.1016/j.str.2008.10.009

**Published:** 2008-12-12

**Authors:** Xue Yuan Pei, Christopher M. Titman, René A.W. Frank, Finian J. Leeper, Ben F. Luisi

**Affiliations:** 1 Department of Biochemistry, University of Cambridge, 80 Tennis Court Road, Cambridge CB2 1GA, UK; 2 Department of Chemistry, University of Cambridge, Lensfield Road, Cambridge CB2 1EW, UK

**Keywords:** PROTEINS

## Abstract

The pyruvate dehydrogenase multienzyme assembly (PDH) generates acetyl coenzyme A and reducing equivalents from pyruvate in a multiple-step process that is a nexus of central metabolism. We report crystal structures of the *Geobacillus stearothermophilus* PDH E1p subunit with ligands that mimic the prereaction complex and the postdecarboxylation product. The structures implicate residues that help to orient substrates, nurture intermediates, and organize surface loops so that they can engage a mobile lipoyl domain that receives the acetyl group and shuttles it to the next active site. The structural and enzymatic data suggest that H128β performs a dual role: first, as electrostatic catalyst of the reaction of pyruvate with the thiamine cofactor; and second, as a proton donor in the second reaction of acetyl group with the lipoate. We also identify I206α as a key residue in mediating the conformation of active-site loops. We propose that a simple conformational flip of the H271α side chain assists transfer of the acetyl group from thiamine cofactor to lipoyl domain in synchrony with reduction of the dithiolane ring.

## Introduction

**Figure 1:**
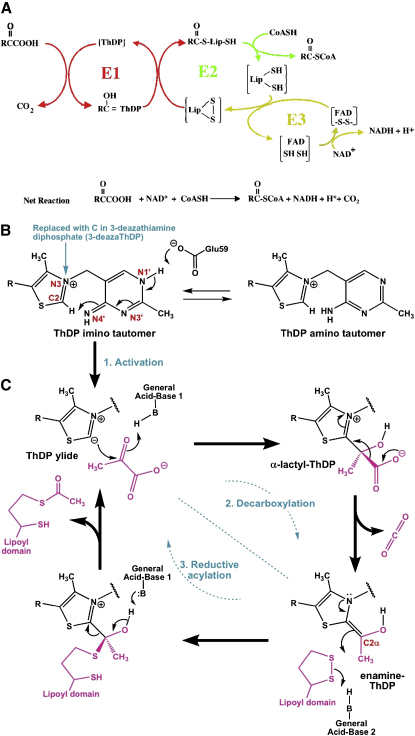
The PDH Catalytic Cycle and the Thiamine Reaction in the E1 Subunit (A) The reaction scheme for PDH and other 2-oxoacid dehydrogenases generally. “R” is a methyl group for the case of PDH. The reactions catalyzed by E1p (pyruvate decarboxylase) are shown in red, E2p (dihydrolipoamide acetyltransferase) in green, and E3 (dihydrolipoamide dehydrogenase) in yellow. E1p catalyzes both the oxidative decarboxylation of pyruvate and the reductive transfer of the acetyl product onto the lipoyl group of the E2p lipoyl domain. E2p catalyzes the transfer of the acetyl group from the lipoyl domain to CoA creating acetyl CoA, and E3 regenerates the disulphide bond in the dithiolane ring of the lipoyl group (see text for further discussion). (B) The thiamine diphosphate cofactor (“R” corresponds to -CH_2_CH_2_OP_2_O_6_^3−^). ThDP bound to E1p adopts the imino tautomer of the aminopyrimidine before forming the activated ylide by deprotonation of its C2 atom. An invariant glutamate (E59β) triggers proton transfer via N4′ and N1′. The substitution of the N3 with carbon in the 3-deazaThDP derivative is indicated. (C) The two half-reactions catalyzed by E1p are decarboxylation and reductive acetylation, in which the first substrate is pyruvate and the second is a lipoyl group attached to a pendant protein domain of E2p. Two general-acid/base catalysts are required. N3′ of ThDP has been proposed to act as the first one and E1p H128β as the second one.

Pyruvate dehydrogenase (PDH) is a multienzyme complex that catalyzes the oxidative transformation of pyruvate to acetyl coenzyme A (CoA), CO_2_, and reducing equivalents in the form of NADH. This challenging reaction is accomplished in a multistage process requiring three different enzymes (schematic summary presented in Figure 1A) (Berg and de Kok, 1997; Chauhan et al., 2000; de Kok et al., 1998; Patel and Harris, 1995a, 1995b; Perham, 1991; Schellenberger, 1998). The first step involves decarboxylation of pyruvate, which proceeds through formation of a covalent intermediate with the thiamine diphosphate cofactor (ThDP) in the active site of the pyruvate decarboxylase subunit (E1p; EC 1.2.4.2). E1p also catalyzes the second reaction step in which the acetyl group is transferred regiospecifically onto one of the sulfur atoms of a lipoyl moiety. The pendant lipoyl group is attached to a mobile domain from the acetyltransferase subunit that transfers the acetyl group onto CoA (E2p; EC 2.3.1.12). The third enzyme, E3, regenerates the dithiolane ring of the lipoate. In common with other members of the 2-oxo acid dehydrogenase family, PDH forms an enormous assembly comprising the three types of subunit (Perham, 2000).

PDH is assembled around a central core formed by multiple copies of E2p that are arranged with either octahedral or icosahedral symmetry (24 or 60 protomers, respectively), depending on organism (Perham, 2000). The crystal structures of the 60-mer E2p cores of *G. stearothermophilus* and *Enterococcus faecalis* reveal an icosahedral shell with an outer diameter of 237 Å, large openings of 52 Å diameter across each of the 12 five-fold symmetry axes, and an inner cavity with a diameter of 118 Å (Izard et al., 1999; Milne et al., 2002). Emanating from this central core are two smaller globular domains connected by linker polypeptide, one of which is the “peripheral subunit binding domain” (PSBD) that interacts with E1p or E3 to support their organization in a concentric spherical shell around the E2p core (Lengyel et al., 2008). Tethered to the PSBD by a ∼50-residue flexible linker is the lipoyl domain that bears lipoate as a covalent modification on a surface-exposed lysine residue. The lipoyl domain is mobile to the extent that it can communicate the acetyl-adduct roughly 90 Å from the E1p to the E2p active site. It traverses this distance again to return the reduced dithiolane to the E3 subunit, where the ring is oxidised and NADH generated. PDH activity can affect the balance between anaerobic and aerobic metabolic pathways of energy generation, and it is accordingly highly regulated in mammals through the activities of a kinase (EC 2.7.1.99) and phosphatase (EC 3.1.3.43) that modify the E1p subunit (Seifert et al., 2007).

The two reactions catalyzed by E1p involve the thiamine diphosphate cofactor (ThDP; Figure 1B). The protein induces a fixed “V” conformation in the ThDP that brings the N4′ amino group of the pyrimidine ring into proximity with the C2 proton of the thiazolium ring (Muller et al., 1993; Kern et al., 1997). In concert with the transfer of the N1′ proton to an invariant glutamate (Figure 1B) (Muller et al., 1993; Kern et al., 1997), the C2 proton is extracted by the N4′ in the imino tautomer to generate the highly reactive carbanion (or “ylide”) (Breslow, 1957). The C2 carbanion makes a nucleophilic attack on the keto carbon of the pyruvate to give a strained α-lactyl-ThDP intermediate (Figure 1C) that eliminates carbon dioxide to form the metastable enamine-ThDP, also known as hydroxyethylidene-ThDP (Arjunan et al., 2006; Jordan, 2003). The enamine-ThDP intermediate reductively acylates the lipoyl group with the likely participation of a general acid from the protein (Fries et al., 2003a). The acetyl-lipoyl adduct (S-acetyldihydrolipoamide) then shuttles to the E2p subunit, and the reaction continues as indicated in the overall scheme summarized in Figure 1A (Perham, 2000).

The E1p subunit of PDH in all known eukaryotes and Gram-positive bacteria is an (α_2_β_2_) heterotetramer, whereas the α− and β−like subunits are fused as a single polypeptide in Gram-negative bacteria, and two of these associate to form homodimers. The α_2_-homodimers and α_2_β_2_-heteroheteromers are structurally similar and each contains two active sites. Because the sites catalyze successive steps with two different substrates, namely pyruvate and lipoate, as described above, E1p displays “ping pong” kinetic behavior. However, the enzyme also displays several unanticipated properties, such as half-of-the-sites reactivity and asymmetry of the exposed surface loops at the entry to the catalytic site (Chauhan et al., 2000). These features might be explained by a “proton wire” model, whereby the active sites communicate through the extended transfer of a single proton from one site to the other (Frank et al., 2004, 2007). This communication establishes the ThDP of one active site as a long-range general-acid/general-base for the other, and vice versa. The proton-wire model also proposes that surface-exposed loops become ordered around the cofactor once it takes on its activated, ylide form. Kinetic data corroborate the nonequivalence of active sites and the synchronization of activation in the human E1p, in support of this proposal (Seifert et al., 2006; Kale et al., 2007). However, the mechanism might not be general to the wider 2-oxoacid dehydrogenase family, because recent data indicate that the subunits do not communicate by this mechanism in the E1b (α-keto acid decarboxylase subunit of the branched chain α-keto acid dehydrogenase) subunit of the human branched chain α-keto acid dehydrogenase (Li et al., 2007).

Insight into the stereochemistry of E1p catalysis has come from crystal structures of the enzyme in complex with analogs of transition states or intermediates. For instance, *Escherichia coli* E1p has been studied in complex with a ThDP derivative that mimics the transient α-lactyl-ThDP intermediate (Arjunan et al., 2006). The pyruvate's carboxylate group is replaced in that analog with a bulky methyl phosphonate that the enzyme cannot remove. The α-lactyl-ThDP analog has a tetrahedral geometry at the Cα position, which is associated with a distortion of the thiazolium ring proposed to facilitate the elimination of the carbon dioxide (Arjunan et al., 2006; Wille et al., 2006). Strain in the α-lactyl-ThDP is also proposed to be enforced by a hydrogen bonding interaction between the ThDP N4′ and the C2α-OH groups (Arjunan et al., 2006). Strained bond geometries in the thiazolium ring have also been proposed to be an important factor in the catalytic path of the ThDP-dependent enzyme transketolase (Asztalos et al., 2007). With the strain-relieving elimination of carbon dioxide, the metastable enamine-ThDP intermediate is formed in E1p.

Two general-acid/general-base groups are required for the transfer of the acetyl group from the enamine-ThDP onto the dithiolane ring of the lipoate (Nemeria et al., 2002; Fries et al., 2003a). The 1′,4′-imino tautomer of ThDP (Figure 1B) can act as one of the general bases for this process (Nemeria et al., 2007a, 2007b). It has been proposed that this 1′,4′-imino tautomeric form is favored in the α-lactyl-ThDP intermediate compared with either the Michaelis complex or the free, activated enzyme (Nemeria et al., 2004). The active-site loops become ordered when the enamine is formed, and that is likely to facilitate interaction with the E2p lipoyl domain and to organize the active site so as to avoid misreactions of the enamine-ThDP, such as the potential reaction with a second pyruvate molecule (Kale et al., 2007; Arjunan et al., 2006; Frank et al., 2004).

To gain further insight into the role of active-site residues of E1p, we have studied crystal structures that mimic different catalytic stages of the reaction cycle. The initial stage of catalysis is represented by the structure of E1p/E2p-PSBD with bound pyruvate. The reaction does not proceed because the cofactor has been replaced with the derivative 3-deazaThDP (thiamine diphosphate derivative where the N3 in the thiazolium ring is replaced with carbon) that mimics the ylide form but is unable to react with substrate. We have also examined the second catalytic stage represented by the structure of E1p with an analog of the enamine-ThDP intermediate, namely 2-(1-hydroxyethyl)-3-deazaThDP (Figure 4A). Each of these structures suggests different catalytic roles for active-site residues (including H128β, Q83α, and others that form active-site loops). To investigate the role of these residues and the active-site loops, mutations of E1p were generated that reveal specific perturbations in one or other of the two catalytic assays. These data define the catalytic role of several residues and coordinated conformational adjustments of the entrance loops of the active site that accompany substrate recognition and catalytic turnover. Finally, these multiple structural snapshots suggest that bound water molecules in the active site act to “gate” substrate recognition of pyruvate, whereas the ordering of the active-site loops gates the recognition of the second substrate, the lipoyl domain. Based on structural modeling, we propose that a simple conformational flip of the side chain of H271α acts as an electrostatic switch that assists the orchestrated transfer of the acetyl group from thiamine cofactor to the lipoyl domain and the reduction of the dithiolane ring.

## Results

### Quaternary Structure of the (α_2_β_2_) Heterotetramer in Complex with the Peripheral Subunit Binding Domain

Using the crystal structure of the (α_2_β_2_) heterotetrameric *G. stearothermophilus* E1p (Frank et al., 2004) (Protein Data Bank [PDB] code 1W85) for molecular replacement, we have solved the structure of the E1p/E2p-PSBD complex with bound 3-deazaThDP and pyruvate at 2.3 Å resolution. This structure represents the early prereaction complex. We have also solved the structure of the E1p/E2p-PSBD complex with bound 2-(1-hydroxyethyl)-3-deazaThDP at 2.5 Å resolution, representing an analog of the postdecarboxylation enamine-ThDP intermediate. Crystal structures were also determined for the E1p active-site mutant with an interesting catalytic defect: I206α to A. Although crystals were also obtained of the mutants H128β to N and H128β to Q, these were lacking the cofactor and so are not presented here; however, they do corroborate the location of the PSBD (see below). All the crystals belong to the same space group and have similar cell dimensions to those of the parent structure (crystallographic and refinement data are summarized in Tables 1 and 2, respectively). The protomers of the refined structures superimpose onto the wild-type enzyme with small root-mean-square deviations of main chain atoms (ranging from 0.1 to 0.6 Å).

**Figure 2:**
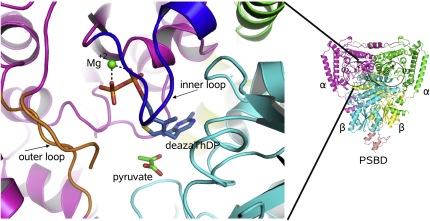
Quaternary Structure of the E1 Subunit and the Adjustable Active-Site Loops The panel on the right shows the heterotetrameric α_2_β_2_ structure of the E1p with the bound PSBD from E2p. The panel on the left shows a zoom into the environment of the active site, showing the inner and outer loops that become structured as predicted (Frank et al., 2004). The figure shows the early prereaction complex comprising enzyme, 3-deazaThDP, and pyruvate (see also Figure 3). The PSBD is shown in salmon pink, the α subunits in magenta and green, and the β subunits in yellow and cyan. A portion of the outer loop had poorly defined electron density and was not included in the model.

The PSBD was not used in the molecular replacement, but well-defined density for this domain was apparent at the same binding site identified in earlier crystallographic studies (Figure 2, right panel) (Frank et al., 2004, 2005). In relation to the wild-type structure, the quaternary structure and PSBD interactions remain unchanged in the intermediate structures of the E1p/E2p-PSBD complex. In agreement with earlier structural and solution studies (Frank et al., 2005; Fries et al., 2003a; Jung et al., 2003; Jung and Perham, 2003), a single PSBD engages one E1p heterotetramer in all crystal structures examined here (Figure 2, right panel). The monomeric PSBD lies on the two-fold molecular symmetry axis of the E1p; consequently, the protein-protein interface is asymmetric (Frank et al., 2005).

### A Structural Analog of the Early Prereaction Complex

**Figure 3:**
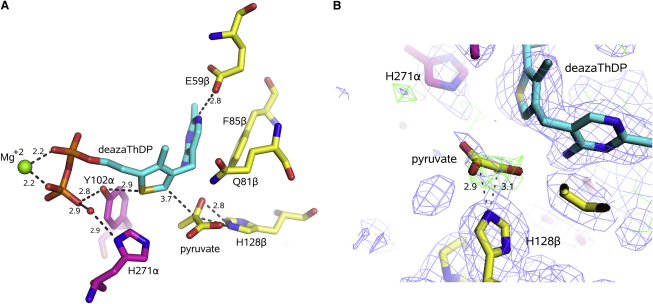
The Pyruvate Complex (A) Pyruvate forms close contacts with H128β. Also shown are other key active site residues, for instance, Y102α, which is pinioned by close polar contacts with the thiazole S (2.9 Å) and a nonesterified oxygen of the terminal phosphate of 3-deazaThDP (2.9 Å). E59β activates the proton extraction shown in Figure 1B, and H271α is proposed to assist the reductive acetylation of the lipoate. F85β makes an aromatic stacking interaction with the pyrimidine ring of the cofactor. The carbon backbone of the pyrimidine and thiazole rings of the 3-deazaThDP is shown in cyan, residues of the α-subunit in purple, residues of β-subunit in yellow, water in the red sphere, Mg^2+^ ion in the green sphere, and pyruvate in yellow (carbon) and red (oxygen). (B) Electron density for the putative pyruvate and surroundings. The map was calculated by omitting the pyruvate before refinement by simulated annealing. The blue map is 2Fo–Fc and the green is positive density above 3σ in the Fo–Fc difference map. Residue F85β to the left of P125β forms part of a hydrophobic embrace of the H128β (see text). The dashed lines are labeled with distances (Å) between the corresponding atoms.

The thiamine diphosphate derivative 3-deazaThDP contains a carbon atom replacing the ring nitrogen at position 3 (Figure 1B) and mimics the activated ylide of the cofactor (Mann et al., 2004). However, one difference from the genuine ThDP ylide is that C2 has a hydrogen atom attached in the 3-deazaThDP derivative. The *G. stearothermophilus* E1p/E2p-PSBD complex was dialyzed extensively against an excess of the 3-deazaThDP in the presence of magnesium ions, which are required to accommodate the diphosphate group of the cofactor in its binding pocket. Pyruvate was soaked for several days into the cocrystals of E1p and 3-deazaThDP, and this resulted in density in the active-site pocket that is likely to be due to pyruvate binding (Figure 3; seeFigure 2 for location with respect to the whole structure). Although four copies of the active site occupy the crystal asymmetric unit, the density for the putative pyruvate is found in only two. In the remaining two sites, well-defined water molecules occupy the corresponding position. Electron density maps were calculated by omitting the pyruvate from the model before refinement by simulating annealing (Figure 3B). It was not possible to definitively assign the orientation of the pyruvate using the electron density alone; however, the location of neighboring polar and nonpolar groups in the active-site pocket reduces the possible orientations. In one preferred orientation, the Cα carbon of the pyruvate is roughly 3.7 Å from C2 of 3-deazaThDP, its potential reaction partner, and is off the plane of the thiazole ring. Although the pyruvate is not in an ideal location or orientation for the reaction, it is unlikely that the displaced location of the pyruvate is entirely a consequence of the steric exclusion by the C2 proton of the 3-deazaThDP, because contacts with the protein support the substrate in this position. Therefore we propose that this structure represents a close analog of a prereaction complex en route to forming the reactive Michaelis complex. The imidazole Nɛ of H128β contacts the pyruvate 2-oxo atom, one of the carboxylate oxygen atoms and the keto carbon of the pyruvate (Figure 3). Notably, the imidazole of H128β is tightly embedded in a hydrophobic nest formed by the nonpolar side chains of P125β, T124β, F85β, and F82β, and this is likely to perturb its pK_a_. The Nδ of H128β contacts the backbone carbonyl of E126β, and although the orientation is not optimal for a hydrogen bond, it is likely that this is the nitrogen that has an attached hydrogen atom to favor the polar contact. Consequently, the imidazole Nɛ does not have a proton and its contact to the pyruvate 2-oxo is unlikely to be a hydrogen-bonding interaction. The interaction with Nɛ of H128β might help to distinguish pyruvate from other carboxylic acids that lack the 2-oxo group. In other 2-oxoacid dehydrogenase E1 subunits, there are geometrically equivalent side chains to H128β that can support a similar interaction and might contribute to substrate recognition.

The ThDP is engaged with the protein through a constellation of polar and nonpolar contacts. None of these interactions changes significantly between the pyruvate-bound structure and the apo structure. The N4′ of the cofactor donates a hydrogen bond to the carbonyl group of I142α and the N3′ accepts a hydrogen bond from the amide NH of I144α. These contacting residues lie in a reverse turn conformation at the end of a helix that orients the peptide backbone to favor these hydrogen-bonding interactions, and we suggest that these bonds are likely to influence the tautomer equilibrium of the amino-pyrimidine ring.

The hydroxyl group of Y102α is wedged between the sulfur atom of the thiazolium ring (2.9 Å) and one of the nonesterified oxygens of the terminal phosphate of the ThDP (2.8 Å) (Figure 3A). The corresponding residue in human E1b, namely Y113α, has been proposed to affect the α-carbanion resonance state of the enamine-ThDP, because the substitution with phenylalanine boosts k_cat_ but renders the enzyme more susceptible to paracatalytic inactivation (Machius et al., 2006). Thus, the close polar contacts of the tyrosine hydroxyl with the thiazole sulfur might repress the full potential of the cofactor in human E1b and the bacterial E1p, but provide protection against aberrant reactions.

### A Mimic of the Postreaction Complex

**Figure 4:**
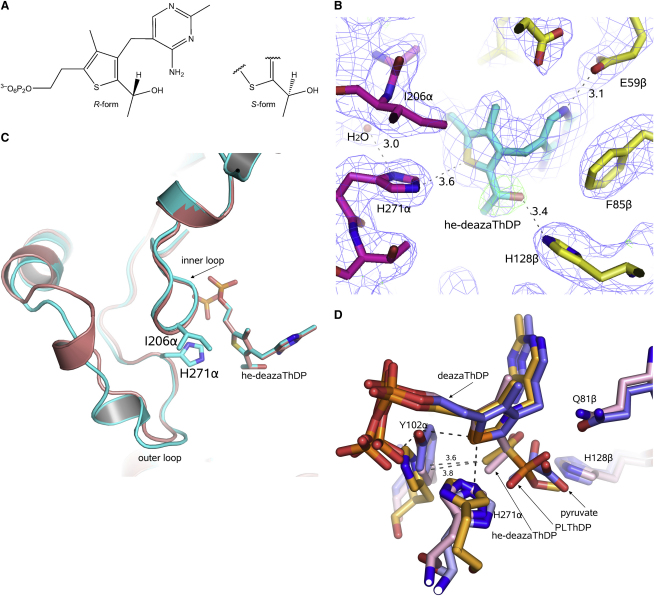
A Mimic of the Enamine-ThDP Postreaction Complex (A) Chemical structure of 2-(1-hydroxyethyl)-3-deazaThDP, abbreviated he-deazaThDP in other figures. The R- and S-configurations are indicated. (B) A view of the 2-(1-hydroxyethyl) group of he-deazaThDP interacting with E1p H128β. The map was calculated by omitting the 2-(1-hydroxyethyl) group before refinement by simulated annealing. The blue map is 2Fo–Fc and the green is positive density above 3σ in the Fo–Fc difference map. (C) A view of the loop region in two superimposed subunits of 2-(1-hydroxyethyl)-3-deazaThDP E1p/E2p-PSBD structure. The protomers are related by noncrystallographic symmetry, and the differences in the conformations of the outer loop can be seen in the overlay. H271α from the outer-loop region and I206α from the inner-loop region are represented in stick format. (D) An overlay of the α-lactyl-ThDP mimic (PLThDP; α-phosphonolactylthiamine diphosphate) bound to *E. coli* E1p (Arjunan et al., 2006) on the active-site pocket of the *G. stearothermophilus* E1p pyruvate complex. The carbon atoms are shown in yellow for the *E. coli* E1p, and pink and gray, respectively, for the *G. stearothermophilus* E1p he-deazaThDP and 3-deazaThDP pyruvate complexes. The overlay suggests that structural reorganizations are likely to accompany the transition from the early pyruvate complex to the predecarboxylation intermediate. If the *G. stearothermophilus* pyruvate complex formed a similar α-lactyl-ThDP intermediate, there would be minor steric clashes with Y102α that could be relieved with small local adjustments.

We prepared cocrystals of *G. stearothermophilus* E1p/E2p-PSBD in complex with 2-(1-hydroxyethyl)-3-deazaThDP (he-3-deazaThDP), an analog of the enamine-ThDP intermediate (Figure 4). The crystals were grown in the presence of a di-domain fragment of E2p encompassing the lipoyl domain, the PSBD and the intervening linker, with the expectation that the lipoyl domain might be captured in a state in which it was bound to E1p. Unfortunately, the crystal structure did not reveal any electron density for the lipoyl domain. Nonetheless, the map has clear density for the he-3-deazaThDP (Figure 4B), and it provides a view of the intermediate preceding the engagement of the lipoyl domain.

The ligand he-3-deazaThDP mimics the postdecarboxylation enamine-ThDP intermediate in both structure and charge state, but with a distorted geometry. The true enamine intermediate is sp^2^ hybridized at C2α, but the C2α atom of our analog is sp^3^ hybridized. Although a racemic mixture was used in the crystallizations (Figure 4A), the electron density map suggests that the *R* enantiomer was preferred in the crystal. The true α-lactyl-ThDP intermediate is thought to have the equivalent configuration (with the CO_2_^−^ group in place of the H) (Arjunan et al., 2006).

In the previous section, we described how H128β contacts the pyruvate. H128β also forms weak hydrogen bonds with he-3-deazaThDP (Figure 4B). The H128β Nɛ atom contributes to a hydrogen-bonding network involving the OA atom of he-3-deazaThDP (distance of 3.4 Å) and the cofactor's N4′ (distance 2.5 Å). The corresponding interactions in the authentic α-lactyl-ThDP intermediate might favor the elimination of carbon dioxide. H128β maintains roughly the same relative position in the structures of the he-3-deaza-ThDP complex and the pyruvate complex (see Figure S1 available online). In principle the H128β side chain could support a second bound pyruvate, but the overlay in Figure 4D suggests that this would generate steric clashes, which in fact could help to prevent possible misreaction of a second pyruvate with the enamine of this enzyme.

### Conformation of Loops at the Active-Site Entrance

Inner and outer loops that cover the active site of E1p are important for the enzyme reaction (Fries et al., 2003b). The loops help stabilize cofactor binding to the E1p and assist in the formation of the enamine intermediate and the transfer of the acetyl group onto the lipoate through their engagement of the shuttling lipoyl domain (Kale et al., 2007, 2008; Perham, 2000). Protease digestion experiments indicate that the loops become structured in the presence of the ylide mimic, 3-deazaThDP (Frank et al., 2004), which suggests that the activation of the cofactor is linked with condensation of the loops. The location of these loops with respect to the quaternary structure is indicated in Figure 2 (left panel).

In the structure of *G. stearothermophilus* E1p/E2-PSBD with ThDP, the inner and outer loops (residues 203α to 212α and 275α to 293α, respectively) are ordered in one subunit, but disordered in the partner subunit (Frank et al., 2004, 2005). We observe that the inner and outer loops are also both ordered in the he-3-deazaThDP complex structure in all four subunits of the asymmetric unit. In the pyruvate-E1p cocrystals, the inner loops are ordered but the outer loops are not, again for all four subunits in the asymmetric unit.

The active-site loops and the ThDP cofactor interact through extensive hydrogen-bonding interactions, hydrophobic interactions, and aromatic ring stacking. The inner loop contacts the diphosphate group of the cofactor and coordinates the Mg^2+^ ion and a hydrated network involving α-subunit residues D173α, N202α, and Q200α (Frank et al., 2004). In both the he-3-deazaThDP and the pyruvate complex, a water-mediated interaction occurs between the inner loop I206α main-chain N atom and O22 atom of the ThDP diphosphate group. These contacts are also seen in the substrate-free structure (Frank et al., 2004, 2005). The H271α Nɛ atom forms a contact with the S atom of the cofactor in the wild-type and 3-deazaThDP structures (distance 3.3Å). This histidine is part of the outer loop that becomes structured with the activation of the cofactor (Frank et al., 2005), and we observe that the loop is ordered in all the multiple copies of the structure with the he-3-deazaThDP bound, although the details of its conformation differ among the copies (Figure 4C). Superimposition shows that H271α in the *G. stearothermophilus* E1p corresponds to H407 in *E. coli* E1p (Figure 4D), which has been shown to play key roles in organization of the active-site loops (Arjunan et al., 2006; Kale et al., 2007) and reductive acetylation of the second substrate (Nemeria et al., 2002).

### Mutational Analysis of Residues in the Catalytic Pocket and Recognition Loops

The crystal structures of the pyruvate-bound state and enamine-ThDP mimic, taken together with results from published studies (Nemeria et al., 2002; Fries et al., 2003a, 2003b), identify residues that are potentially important for substrate recognition, for binding the transition states (H128β, H271α), for supporting conformational changes of active-site loops (I206α), and for providing general-base functionality (H271α or H128β). We evaluated the effects of some of these residues by generating several new site-directed mutations.

The catalytic activities of the mutants and wild-type were assessed by the colorimetric reaction with the artificial electron acceptor, dichlorophenolindophenol (DCPIP), which in the wild-type enzyme is probably limited by accessibility of DCPIP to the active site, but in low-activity mutants might give an indication of the rate of enamine formation. A schematic of the DCPIP reaction is provided in Experimental Procedures. The E1p active-site mutants (H128Nβ, H128Qβ, I206Aα, and Q81Eα) do not affect binding to E2p (Figure S2), indicating that they can be assembled into the PDH complex. The activity of the fully reconstituted PDH complex was assayed by formation of NADH.

**Figure 5:**
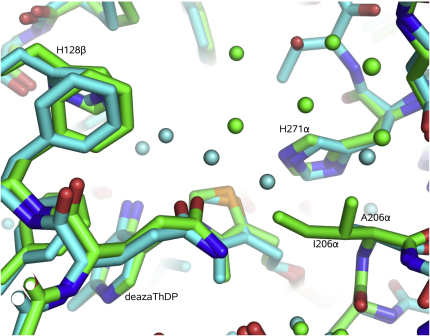
Hydration Patterns in the Active-Site Pocket Near the ThDP Cofactor The substitution of the bulky isoleucine by alanine in the I206Aα mutant changes the hydration pattern around the cofactor. The wild-type structure (green) is overlayed with the mutant in complex with 3-deazaThDP (cyan).

I206α contacts the thiazolium ring of the ThDP cofactor (Figure 4C). The crystal structure of the mutant in which this residue is replaced with alanine reveals changes in the hydration pattern within the active-site pocket, with water molecules now closer to the thiazole group of the cofactor (Figure 5). This mutant has much greater DCPIP activity than the wild-type enzyme (Table 3), which might be attributed to the greater access to the pocket. In contrast to the boost in DCPIP activity, this mutant showed impaired PDH complex activity, in which the rate of reductive acetylation is the rate-limiting step (Fries et al., 2003b).

H128β has been proposed to be a proton donor during the reductive transfer of the acetyl group to lipoate (Fries et al., 2003a). To test this role, we examined the consequences of comparatively conservative substitutions of this residue with Q or N on the activity of the enzyme. The H128Qβ mutant has nearly the same activity as the wild-type E1p in the DCPIP assay, but in striking contrast, the H128Nβ mutant has dramatically reduced activity (Table 3). The PDH complex activity of both these mutants was less than 5% that of the wild-type complex, with the H128Nβ mutant being consistently lower than that of the H128Qβ mutant.

Although both H128Nβ and H128Qβ mutants were crystallized in the presence of 3-deazaThDP, the crystal structures reveal that the cofactor analog is absent from the catalytic pocket, so it is not possible to comment on the structural consequences of the mutations in the transition states. The structure of *E. coli* E1p lacking the cofactor reveals a network of water molecules occupying the ThDP pocket (Chandrasekhar et al., 2006). A similar effect is seen here for the two apo-structures (not shown).

### Modeling of Engaged Lipoate

**Figure 6:**
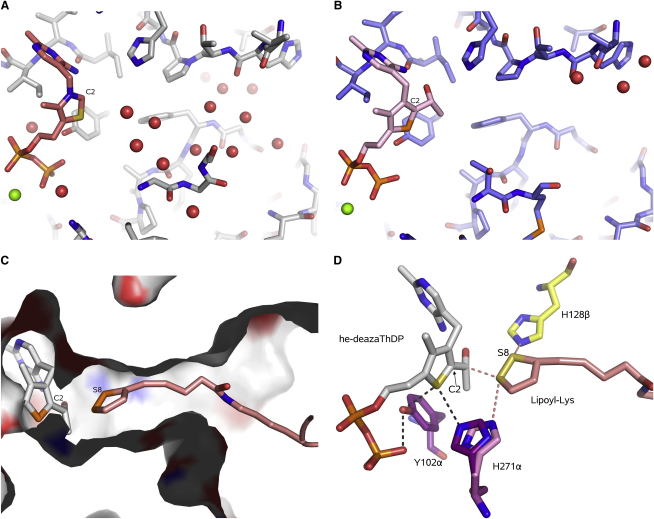
Postulated Path of the Lipoate in the E1 Active Site (A) Hydration pattern along the pathway to the active site. The enzyme exterior is on the right in this view. (B) Hydration pattern in the lipoate path in the he-3-deazaThDP complex. The red spheres represent the water molecules. (C) Model of the docked oxidised lipoate. The access channel is shown as a section through a space-filling surface. The cofactor, lipoyl-lysine, and a portion of the lipoyl domain peptide backbone are shown. (D) A speculative model showing how the dithiolane ring might be presented to the active-site pocket residues in E1p. A simple 180° flip about the Cγ–Cβ bond brings the H271αNɛ away from close contact with the thiazole S and orientated toward the thiolane ring of the oxidised ring. This could orchestrate electronic perturbations of the thiazole and dithiolane rings that facilitate the reductive acylation.

An extensive hydration pattern occludes access to the active site in the crystal structures of the native E1p with bound ThDP (Figure 6A). These water molecules must be displaced to accommodate the incoming lipoate for the second stage of the catalytic cycle (Aevarsson et al., 1999; Perham, 2000). In the crystal structure of the he-3-deazaThDP, the pocket has few apparent fixed water molecules (Figure 6B; see also Figure S3).

Earlier modeling of the lipoate docked into the active site suggested key contacts with active-site residues that might assist the reduction of the dithiolane ring. To reach the thiamine, the lipoylated-lysine arm must fully stretch from the lipoyl domain, and the E2 lipoyl domain must engage the E1 to deliver the lipoyl group to the site (Jones and Perham, 2008; Jones et al., 2001; Perham, 2000). A funnel-shaped region between the interface of E1α and E1β subunits provides access to the active site. In the 3-deazaThDP structure, this region is filled with water, but as mentioned above, the waters are not ordered in the he-3-deazaThDP structure. It is likely that those waters are displaced during the formation of the Michaelis and strained enamine-ThDP complexes.

Lipoylated-lipoyl domain was docked into the he-3-deazaThDP structure. The thiazole C2 and lipoate S distance was used as a restraint in the modeling. After manual adjustment, the model was energy minimized. The model accommodates the dithiolane ring and aliphatic portion in the channel leading to the active site without steric clash and suggests that H128β and H271β are both in a good orientation to support the acetyl transfer (Figure 6C). Like Y102α mentioned earlier, H271α might electronically modulate the enamine-ThDP through the close-contact with the thiazole S atom, and our docking model suggests that simply flipping 180° about the Cγ-Cβ bond brings the H271α Nɛ away from the close contact with the thiazole S and orientated toward the thiolane ring of the oxidised ring (Figure 6D).

## Discussion

The E1p enzyme from pyruvate dehydrogenase is a prototypical thiamine-dependent decarboxylase subunit from the extensive family of 2-oxo acid dehydrogenases (Perham, 2000). In this study we have determined the crystal structures of *G. stearothermophilus* E1p/E2-PSBD bearing analogs of catalytic intermediates and active-site mutants. The crystal structures figuratively provide snapshots of two stages in the E1p catalytic mechanism.

### Structure of the Pyruvate Complex

One key residue of the catalytic pocket, implicated in both cofactor binding and catalysis, is H128β. An earlier study found that substituting H128β for alanine had a large inhibitory effect on the reaction with the artificial electron acceptor DCPIP and inactivated PDH activity (Fries et al., 2003a). In this study, we made the more conservative substitutions of H128β to the polar residues asparagine and glutamine (H128Nβ and H128Qβ) to further explore the role of this residue. The H128Qβ mutation has the same activity as wild-type enzyme in the DCPIP assay, suggesting that the rate of enamine-ThDP formation is not dramatically affected by the H128Qβ substitution. However, replacement of H128β with the shorter polar residue asparagine has a profound effect on activity (Table 3), similar to that of the H128Aβ mutation (Fries et al., 2003a).

The question arises why substitution of H128β by glutamine is effectively neutral in the DCPIP assay, whereas substitution with asparagine or alanine is deleterious. Glutamine and asparagine differ only by a single methylene group, so their functional dissimilarity reflects a requirement for precise positioning during catalysis. A further indication that the residue at position 128β plays a defined role is provided by the crystal structure of the pyruvate complex in the wild-type enzyme, which suggests that H128β can form a polar interaction with the substrate. The interaction is likely to steer and orient the keto carbon of the pyruvate for presentation to the reactive carbanion of the activated ThDP ylide, and the close polar interaction might help to activate the substrate. Because glutamine can be a hydrogen-bond donor or acceptor, it is expected that H128Qβ could also make a stabilizing interaction with substrate. Asparagine, however, would be too short, possibly accounting for its deleterious effect in the DCPIP assay. Because glutamine will substitute as well as histidine, we suggest that H128β does not serve as a general-acid/general-base for the first stage of the reaction; namely, the formation of the enamine intermediate.

Although H128Qβ is a neutral substitution in the context of the decarboxylation step, it is inferred to be severely impairing in the step of lipoate acetylation (Table 3). H128β might serve as a general-acid/base during the reductive acetylation of the lipoate (general-acid/base 2 in Figure 1C), which is the rate-limiting step of the reaction (Perham, 2000; Jones et al., 2001). Although a histidine is conserved in this position in all known E1s, glutamine and asparagine are found in the corresponding position to H128β in some members of the wider family of ThDP-dependent enzymes (Frank et al., 2007). The question therefore arises as to how the glutamine and asparagine substitutions are accommodated in other enzymes because they seem to be disruptive mutations in the context of the *G. stearothermophilus* E1p. First, we suggest that the residue in this position must function as an electrostatic catalyst during the first step of catalysis of all ThDP-dependent enzymes. Asparagine might be accommodated in certain enzymes, but we anticipate that compensating structural changes occur in the active-site pocket to enable the side chain to approach the substrate. Second, we suggest that the residue equivalent to 128β has a second function as a proton donor in the second half of catalysis of all E1, and these enzymes will therefore all require histidine; this function is not universally required in ThDP-dependent enzymes.

Our structural data suggest that a conformational adjustment might be required to accommodate the transition from the substrate-bound complex to the predecarboxylation product. As we discussed earlier, the pyruvate complex is not in an optimal geometry for the ThDP C2 to attack the pyruvate, and it is clear that either the substrate would have to be reorientated, or the thiazolium ring would have to tip substantially. Once formed, it is likely that the α-lactyl-ThDP intermediate would itself also require adjustment to be sterically accommodated. This can be most clearly seen in the comparison of our pyruvate-E1p complex and the *E. coli* E1p predecarboxylation mimic (Figure 4D), which have been superimposed in the reference frame of the thiazole ring. Most strikingly, the α-lactyl-ThDP methyl substituent would have minor steric clashes with Y102α in this common reference frame (the corresponding residues in the *E. coli* enzyme is a histidine). Mechanical strain might accompany the formation of the predecarboxylation state, and this is dissipated in the formation of the postdecarboxylation product. We thus envisage a “spring loaded” mechanism that aids catalysis through the dissipation of the mechanical strain generated as the reaction proceeds to make the first covalent intermediate.

### Conformations of the Active-Site Loops

Structural change frequently accompanies substrate binding in the active sites of enzymes, and a cogent example is the closure of loops decorating the active-site entrance in E1p. The ordering of these loops is probably required for engagement of the lipoyl domain to bring the second substrate into the active-site pocket and helps to synchronize the catalytic steps (Nakai et al., 2004; Kale et al., 2008). Loop organization might also prevent side reactions with a second pyruvate molecule or acetaldehyde (Kale et al., 2007). Active-site entry loops become ordered in the *E. coli* E1p when bound with a mimic of the α-lactyl-ThDP predecarboxylation intermediate state (Arjunan et al., 2006). Although the *E. coli* and *G. stearothermophilus* enzymes do differ in details of their folds and the active-site loops occur in different parts of the overlaid structures, they share in common a disorder-order transition for these loops with certain states of the cofactor. The loops are proposed to become ordered when the ThDP becomes the activated ylide form in the *G. stearothermophilus* structure (Frank et al., 2004). The compound 3-deazaThDP, which has a neutral ring in place of the positively charged thiazolium ring of ThDP, mimics the activated ylide form of the cofactor (Mann et al., 2004) and stabilizes the inner loop in the E1p (Frank et al., 2004). In the structures that we report here, the inner loop and outer loop are both well ordered in the pyruvate-bound and he-3-deazaThDP complexes of E1p. The ordering of the loops in the substrate-bound structure was predicted based on the structure of the predecarboxylation covalent intermediate of the *E. coli* E1p structure (Arjunan et al., 2006). The gain of order in the loops might account for the noted alteration of proton spin relaxation rates for E1p in the presence of lipoyl domain and pyruvate (Jones et al., 2001).

The crystal structure of the complex of 3-deazaThDP with the *G. stearothermophilus* E1p I206Aα mutant reveals that active-site loops (residues α204–209 and α268–290) are ordered. The activity of the I206Aα mutant is several-fold greater than that of the wild-type enzyme in the DCPIP assay, but its PDH activity is only about a third that of the wild-type enzyme (Table 3). A qualitatively similar effect was reported for the H271Aα *G. steraothermophilus* E1p mutant (Fries et al., 2003b) and for the corresponding *E. coli* E1p mutant (H407A) (Nemeria et al., 2002). The differences between the DCPIP and PDH assay activities for these mutants might reflect their differing roles in the first and the second steps of catalysis. Both the I206Aα and H271Aα mutants might provide greater access to the active site, but both might cause inappropriate conformations of the outer loop, so that it no longer engages the lipoyl domain for acyl transfer.

### Structure of the ThDP Enamine-like Complex

The crystal structure of the E1p with bound he-3-deazaThDP provides a view of the catalytic pocket in the enamine-ThDP state. In the true enamine intermediate, the C2α would be coplanar with the thiazole ring in the absence of external steric strain. However, it has been suggested that distortion of the enamine from planarity occurs in certain ThDP-dependent enzymes and this gives the α-carbon more anionic character and thus makes it more reactive (Berthold et al., 2007). Thus, he-3-deazaThDP, in which C2α is tetrahedral, might be a close mimic of the enzyme-bound form of the enamine-ThDP. Although the channel leading to the active-site pocket is well hydrated in the pyruvate complex and the substrate-free state, it has little apparent structured water in the enamine-like state (Figure 6; Figure S3). The displacements of these waters might help to accommodate the lipoate for the next stage of the catalytic cycle. The enzyme thus shows “water-gated” substrate recognition of pyruvate, and “polypeptide loop-gated” recognition of the second substrate, the lipoyl domain.

We also suggest that the reductive acetylation step involves general-acid/general-base catalysis involving H128β, and that H271α might electronically modulate the enamine-ThDP through the close-contact made by its Nɛ atom with the thiazole S atom. Our docking model suggests that by simply flipping about the Cβ-Cγ bond, the H271α Nɛ is brought away from the thiazole and orientated toward the dithiolane ring of the oxidised lipoyl group (Figure 6D). In one of the four asymmetric unit copies of the he-deazaThDP complex, the H271α appears to have made this flip, and this is correlated with the loss of the water molecule that links the side chain to the terminal phosphate of the ThDP. The simple dynamic flip of the side-chain orientation could thus simultaneously perturb the electronic structure of the enamine to favor reaction and support the reduced thiolane ring for orchestrated transfer of the acetyl group. This flip-switch model can account for the catalytic defects caused by substitutions of the histidine in the E1p of *G. stearothermophilus* (Fries et al., 2003a) and *E. coli* (Nemeria et al., 2002).

## Experimental Procedures

### Mutagenesis, Protein Expression, and Purification

Expression vectors encoding the *G. stearothermophilus* E1 mutants I206Aα, Q81Eβ, H128Nβ, and H128Qβ were generated from the pKBstE1a and pKBstE1b plasmids, which express E1α and E1β, respectively (Lessard, 1995), using the Quickchange PCR mutagenesis method and were corroborated by sequencing. The following primers were used to mutate amino acids in the E1 plasmids (mutated bases are underlined):

I206Aα-F 5′CAACCGCTTTGCCGCCTCGACGCCGGTCG,
I206Aα-R 5′CGACCGGCGTCGAGGCGGCAAAGCGGTTG,
Q81Eα-F 5′CCGACCGCCGGGGAGGAAGCGAGCC,
Q81Eα-R 5′GGCTCGCTTCCTCCCCGGCGGTCGG,
H128Qβ 5′CATACGCCGGAGTTGCAATCAGACAGCTTGG,
H128Qβ-R 5′CCAAGCTGTCTGATTGCAACTCCGGCGTATG,
H128Nβ-F 5′CATACGCCGGAGTTGAACTCAGACAGCTTGG
and
H128Nβ-R 5′CCAAGCTGTCTGAGTTCAACTCCGGCGTATG.

Mutant proteins were expressed in TG1 *recO* strain of *E. coli* [K12, Δ(*lacproAB*), *sup*E, *thi*, *hsdΔ*5, *recO*::Tn5 Kanr/F`*traD*36, *proA*^+^B^+^, *laqI*^q^, *lacZΔ*M15] in LB media (containing 100 μg/ml ampicillin and 15 μg/ml kanamycin). *E. coli* strains expressing E1α and E1β were harvested, combined, and lysed together. The heterotetrameric E1 spontaneously self-assembled and was purified by anion-exchange (Q-Sepharose) and size-exclusion chromatography (Superdex-200) as described previously (Lessard, 1995; Lessard et al., 1996). The purity of these proteins was assessed using the NuPAGE SDS-PAGE system (Invitrogen).

### Enzymatic Activity Assays

#### DCPIP Assay

The mutant and wild-type E1p proteins were evaluated for catalytic activity, specifically oxidative decarboxylation. The rate of reduction of the artificial electron acceptor 2,6-dichlorophenolindophenol (DCPIP) by E1p was followed spectroscopically at 600 nm and 30°C to probe the decarboxylation reaction (Khailova et al., 1977; Lessard and Perham, 1994):

$$\text{Elp}\text{-}\text{ThDP}+\text{pyruvate}\to \text{Elp}\text{-}\text{enamine}\text{-}\text{ThDP}+{\text{CO}}_{2}$$

$$\text{Elp}\text{-}\text{enamine}\text{-}\text{ThDP}+\text{DCPIP}+{\text{H}}_{2}\text{O}\to \text{Elp}\text{-}\text{ThDP}+\text{DCPIP}{\text{H}}_{2}+\text{acetate}$$

#### PDH Assay

Pyruvate dehydrogenase complex activity was assayed following the rate of NADH production monitored spectrophotometrically at 340 nm and 30°C following assembly of the PDH, using lipoylated E2 with about a 3-fold excess of E1 and E3. Fifty picomoles E2 was used and the reaction was initiated by the addition of pyruvate and CoA (Danson and Perham, 1976).

### Assay for Interaction of E1 and the E2 Peripheral Subunit Binding Domain

One hundred picomoles *G. stearothermophilus* E1 was incubated with 200 pmol *G. stearothermophilus* E2 di-domain, which comprises the lipoyl domain, the PSBD, and the intradomain linker. The complexes were resolved by native gel electrophoresis using 8% polyacrylamide Tris-glycine buffered gels (Invitrogen) at 80 V for 3 hr (Figure S2).

### Crystallizations

The enamine-ThDP mimic used for cocrystallizations is the compound 2-(1-hydroxyethyl)-3-deazaThDP (he-3-deazaThDP). The preparation of this derivative is described in Leeper et al. (2005). The Cα is a chiral center (Figure 4A), and the racemic mixture was used to prepare cocrystals of he-3-deazaThDP E1/E2 PSBD. A solution of E1/E2 PSBD complex at 10 mg/ml in 20 mM potassium phosphate (pH 7.0) and 0.02% sodium azide was incubated with 5 mM he-3-deazaThDP and 5 mM MgCl_2_ on ice for 2–3 hr. The solution was then mixed in 1:1 volume ratio with crystallization buffer consisting of 8%–12% w/v monomethyl ether polyethylene glycol (MME PEG) 5000 and 0.1 M Na maleate pH 5.5, and the droplet was left to equilibrate against a reservoir of neat crystallization buffer.

The crystals of the pyruvate complex were prepared with the nonreactive ThDP analog 3-deazaThDP (Mann et al., 2004). Crystals of E1 (α_2_β_2_)/E2 PSBD complexed with 3-deazaThDP were obtained using set-drop vapor diffusion by the same approach described above. The crystals were then soaked in mother liquor with 10 mM pyruvate for 1 week and then harvested for data collection.

All mutant crystals were obtained by sitting-drop vapor diffusion using the following condition: 10%–15% w/v PEG 4K, 0.2 M imidazole malate (pH 5.0) in the presence of 5 mM 3-deazaThDP.

Crystals were cryoprotected before flash freezing using crystallization buffer diluted with 20% v/v 2-methyl-2,4-pentanediol and 10% v/v glycerol.

### Structure Determination, Refinement, and Modeling

Diffraction data were collected at 100 K at synchrotron sources and were processed with MOSFLM/SCALA (Leslie, 1992; CCP4, 1994). Crystallographic data are summarized in Table 1. The five structures presented here were determined by molecular replacement using the *G. stearothermophilus* heterotetrameric (α_2_β_2_) pyruvate decarboxylase (E1) (PDB code 1w85) as a search model in PHASER (McCoy et al., 2007). The ligands were removed from the search model: the ThDP, the PSBD, water, magnesium, and potassium. Following rigid-body refinement of this stripped model, the structure was refined by simulated annealing using CNS 1.1 (Brunger et al., 1998) followed by REFMAC refinement (Murshudov et al., 1997) with noncrystallographic symmetry restraints and translation-libration-screw disorder parameters. The models were built using the COOT program (Emsley and Cowtan, 2004) from the CCP4 suite (CCP4, 1994). The refinement parameters for the he-3-deazaThDP molecule were generated with PRODRGS (Schuttelkopf and van Aalten, 2004). The ligand was modeled at the late stages of refinement, and it was corroborated by simulating annealing omit maps calculated by slow-cooling from 3000 K. A summary of the crystallographic refinement parameters is presented in Table 2.

The lipoated lipoyl domain was docked into the E1p structure using the program GRAMM (Vakser, 1996).

## Figures and Tables

**Table 1:** Statistics of X-Ray Diffraction Data

	2-(1-hydroxyethyl)-3-deazaThDP E1p/E2p-PSDB	Pyruvate + 3-deazaThDP E1p/E2p-PSBD	E1p I206Aα
Space group	P2_1_	P2_1_	P2_1_
Unit cell (Å,°)	a = 69.25	a = 68.31	a = 68.67
	b = 231.99	b = 232.82	b = 232.29
	c = 92.61	c = 92.01	c = 91.94
	β = 90.74	β = 91.83	β = 91.2
Resolution (Å)	66-2.5	85-2.3	77.38-2.35
Wavelength (Å) and synchrotron station	0.9795	0.9794	0.933
	ESRF ID23-1	ESRF ID29	ESRF ID14-2
Heterotetramers in asymmetric unit	2	2	2
Number of observations (unique)	369,035	474,043	499,557
	(99,540)	(110,903)	(117,919)
Completeness (%)	98.9 (98.9)	87.5 (87.5)	98.9 (98.9)
Rmerge	0.111 (0.26)	0.097 (0.348)	0.080 (0.227)
<I> /sd	9.1 (5.4)	9.0 (4.7)	13.9 (5.9)
I/sigma	3.1 (2.6)	6.2 (1.9)	7.1 (3.1)
Multiplicity	3.7 (3.7)	4.3 (2.5)	4.2 (3.4)
Wilson B-factor (Å^2^)	46.88	24.69	30.91

**Table 2:** Crystallographic Refinement Data for the *Geobacillus stearothermophilus* E1p/E2p-PSBD Complexes

Complex	Resolution (Å)	R_free_	R_work_	R_free+work_	B-factor (Å^2^)	rms Bond Length (Å) Bond Angle(°) Chiral Restraint (Å^3^)	Ramachandran Plot (% Favored/Allowed/Generously Allowed/Not Allowed)	Cofactor	No. of Water Molecules
E1p/E2p-PSBD he-3-deazaThDP	50-2.5	0.264	0.193	0.197	42.2	0.004/0.748/0.092	90.3/8.2/0.7/0.0	he-3-deaza-ThDP	434
E1p/E2p-PSBD pyruvate + 3-deazaThDP	68-2.3	0.265	0.189	0.193	28.5	0.004/0.736/0.114	90.7/8.5/0.9/0.0	3-deaza ThDP	1053
E1p/E2p-PSBD I206Aα 3-deazaThDP	77-2.35	0.247	0.184	0.184	29.2	0.008/1.17/0.098	90.1/8.6/0.9/0.0	3-deaza ThDP	1138

**Table 3:** Relative Activities of E1p Mutants

Proteins	DCPIP Activity		PDH Complex Activity	
	Activity	SD	Activity	SD
Wild-type E1p	100.0	8.0	100.0	8.5
I206Aα	1331.2	18.9	35.1	8.0
Q81Eα^a^	127.1	3.7	96.8	3.8
H128Qβ	100.2	10.9	3.8	2.4
H128Nβ	0.9	3.4	0.7	0.2

a This is a neutral substitution for comparison (see Supplemental Data).

## Accession Numbers

Refined models and experimental structure factors have been deposited with the Protein Structural Database with the accession numbers 3DUF for the he-3-deazaThDP structure, 3DV0 for the 3-deazaThDP + pyruvate structure, and 3DVA for the I206A structure.

## Supplemental Data

Document S1. Supplemental Experimental Procedures, Supplemental References, and Three Figures

## References

[bib1] Aevarsson A., Seger K., Turley S., Sokatch J.R., Hol W.G. (1999). Crystal structure of 2-oxoisovalerate and dehydrogenase and the architecture of 2-oxo acid dehydrogenase multienzyme complexes. Nat. Struct. Biol..

[bib2] Arjunan P., Sax M., Brunskill A., Chandrasekhar K., Nemeria N., Zhang S., Jordan F., Furey W. (2006). A thiamin-bound, pre-decarboxylation reaction intermediate analogue in the pyruvate dehydrogenase E1 subunit induces large scale disorder-to-order transformations in the enzyme and reveals novel structural features in the covalently bound adduct. J. Biol. Chem..

[bib3] Asztalos P., Parthier C., Golbik R., Kleinschmidt M., Hubner G., Weiss M.S., Friedemann R., Wille G., Tittmann K. (2007). Strain and near attack conformers in enzymic thiamin catalysis: X-ray crystallographic snapshots of bacterial transketolase in covalent complex with donor ketoses xylulose 5-phosphate and fructose 6-phosphate, and in noncovalent complex with acceptor aldose ribose 5-phosphate. Biochemistry.

[bib4] Berg A., de Kok A. (1997). 2-Oxo acid dehydrogenase multienzyme complexes. The central role of the lipoyl domain. Biol. Chem..

[bib5] Berthold C.L., Toyota C.G., Moussatche P., Wood M.D., Leeper F.J., Richards N.G.J., Lindqvist Y. (2007). Crystallographic snapshots of oxalyl-CoA decarboxylase give new insights into catalysis by non-oxidative ThDP-dependent decarboxylases. Structure.

[bib6] Breslow R. (1957). Rapid deuterium exchange in thiazolium salts. J. Am. Chem. Soc..

[bib7] Brunger A.T., Adams P.D., Clore G.M., DeLano W.L., Gros P., Grosse-Kunstleve R.W., Jiang J.S., Kuszewski J., Nilges M., Pannu N.S. (1998). Crystallography & NMR system: A new software suite for macromolecular structure determination. Acta Crystallogr. D Biol. Crystallogr..

[bib8] CCP4 (Collaborative Computational Project, Number 4) (1994). The CCP4 suite: programs for protein crystallography. Acta Crystallogr. D Biol. Crystallogr..

[bib9] Chandrasekhar K., Arjunan P., Sax M., Nemeria N., Jordan F., Furey W. (2006). Active-site changes in the pyruvate dehydrogenase multienzyme complex E1 apoenzyme component from Escherichia coli observed at 2.32 A resolution. Acta Crystallogr. D Biol. Crystallogr..

[bib10] Chauhan H.J., Domingo G.J., Jung H.I., Perham R.N. (2000). Sites of limited proteolysis in the pyruvate decarboxylase component of the pyruvate dehydrogenase multienzyme complex of Bacillus stearothermophilus and their role in catalysis. Eur. J. Biochem..

[bib11] Danson M.J., Perham R.N. (1976). Evidence for two lipoic acid residues per lipoate acetyltransferase chain in the pyruvate dehydrogenase multienzyme complex of *Escherichia coli*. Biochem. J..

[bib12] de Kok A., Hengeveld A.F., Martin A., Westphal A.H. (1998). The pyruvate dehydrogenase multi-enzyme complex from Gram-negative bacteria. Biochim. Biophys. Acta.

[bib13] Emsley P., Cowtan K. (2004). Coot: model-building tools for molecular graphics. Acta Crystallogr. D Biol. Crystallogr..

[bib14] Frank R.A., Titman C.M., Pratap J.V., Luisi B.F., Perham R.N. (2004). A molecular switch and proton wire synchronize the active sites in thiamine enzymes. Science.

[bib15] Frank R.A., Pratap J.V., Pei X.Y., Perham R.N., Luisi B.F. (2005). The molecular origins of specificity in the assembly of a multienzyme complex. Structure.

[bib16] Frank R.A., Leeper F.J., Luisi B.F. (2007). Structure, mechanism and catalytic duality of thiamine-dependent enzymes. Cell. Mol. Life Sci..

[bib17] Fries M., Jung H.I., Perham R.N. (2003). Reaction mechanism of the heterotetrameric (α2β2) E1 component of 2-oxo acid dehydrogenase multienzyme complexes. Biochemistry.

[bib18] Fries M., Chauhan H.J., Domingo G.J., Jung H.I., Perham R.N. (2003). Site-directed mutagenesis of a loop at the active site of E1 (α_2_β_2_) of the pyruvate dehydrogenase complex. A possible common sequence motif. Eur. J. Biochem..

[bib19] Izard T., Aevarsson A., Allen M.D., Westphal A.H., Perham R.N., de Kok A., Hol W.G. (1999). Principles of quasi-equivalence and Euclidean geometry govern the assembly of cubic and dodecahedral cores of pyruvate dehydrogenase complexes. Proc. Natl. Acad. Sci. USA.

[bib20] Jones D.D., Perham R.N. (2008). The role of loop and beta-turn residues as structural and functional determinants for the lipoyl domain from the *Escherichia coli* 2-oxoglutarate dehydrogenase complex. Biochem. J..

[bib21] Jones D.D., Stott K.M., Reche P.A., Perham R.N. (2001). Recognition of the lipoyl domain is the ultimate determinant of substrate channelling in the pyruvate dehydrogenase multienzyme complex. J. Mol. Biol..

[bib22] Jordan F. (2003). Current mechanistic understanding of thiamin diphosphate-dependent enzymatic reactions. Nat. Prod. Rep..

[bib23] Jung H.I., Cooper A., Perham R.N. (2003). Interactions of the peripheral subunit-binding domain of the dihydrolipoyl acetyltransferase component in the assembly of the pyruvate dehydrogenase multienzyme complex of *Bacillus stearothermophilus*. Eur. J. Biochem..

[bib24] Jung H.I., Perham R.N. (2003). Prediction of the binding site on E1 in the assembly of the pyruvate dehydrogenase multienzyme complex of *Bacillus stearothermophilus*. FEBS Lett..

[bib25] Kale S., Arjunan P., Furey W., Jordan F. (2007). A dynamic loop at the active center of the Escherichia coli pyruvate dehydrogenase complex E1 component modulates substrate utilization and chemical communication with the E2 component. J. Biol. Chem..

[bib26] Kale S., Ulas G., Song J., Brudvig G.W., Furey W., Jordan F. (2008). Efficient coupling of catalysis and dynamics in the E1 component of *Escherichia coli* pyruvate dehydrogenase multienzyme complex. Proc. Natl. Acad. Sci. USA.

[bib27] Kern D., Kern G., Neef H., Tittmann K., Killenberg-Jabs M., Wikner C., Schneider G., Hubner G. (1997). How thiamine diphosphate is activated in enzymes. Science.

[bib28] Khailova L.S., Bernkhardt R., Khiubner G. (1977). Study of the kinetic mechanism of the pyruvate-2,6-dichlorophenolindophenol reductase activity of muscle pyruvate dehydrogenase. Biokhimiia.

[bib29] Leeper F.J., Hawksley D., Mann S., Perez Melero C., Wood M.D. (2005). Studies on thiamine diphosphate-dependent enzymes. Biochem. Soc. Trans..

[bib30] Lengyel J.S., Stott K.M., Wu X., Brooks B.R., Balbo A., Schuck P., Perham R.N., Subramaniam S., Milne J.L. (2008). Extended polypeptide linkers establish the spatial architecture of a pyruvate dehydrogenase multienzyme complex. Structure.

[bib31] Leslie A.G.W. (1992). Recent changes to the MOSFLM package for processing film and image plate data. Joint CCP4 + ESF-EAMCB Newsletter on Protein Crystallography.

[bib32] Lessard, I.A. (1995). Protein-protein interaction and the molecular self-assembly of the pyruvate dehydrogenase multienzyme complex. PhD thesis. Cambridge University, Cambridge.

[bib33] Lessard I.A., Perham R.N. (1994). Expression in *Escherichia coli* of genes encoding the E1α and E1β subunits of the pyruvate dehydrogenase complex of *Bacillus stearothermophilus* and assembly of a functional E1 component (α_2_β_2_) *in vitro*. J. Biol. Chem..

[bib34] Lessard I.A., Fuller C., Perham R.N. (1996). Competitive interaction of component enzymes with the peripheral subunit-binding domain of the pyruvate dehydrogenase multienzyme complex of *Bacillus stearothermophilus*: kinetic analysis using surface plasmon resonance detection. Biochemistry.

[bib35] Li J., Machius M., Chuang J.L., Wynn R.M., Chuang D.T. (2007). The two active sites in human branched-chain alpha-keto acid dehydrogenase operate independently without an obligatory alternating-site mechanism. J. Biol. Chem..

[bib36] Machius M., Wynn R.M., Chuang J.L., Li J., Kluger R., Yu D., Tomchick D.R., Brautigam C.A., Chuang D.T. (2006). A versatile conformational switch regulates reactivity in human branched-chain α-ketoacid dehydrogenase. Structure.

[bib37] Mann S., Perez Melero C., Hawksley D., Leeper F.J. (2004). Inhibition of thiamin diphosphate dependent enzymes by 3-deazathiamin diphosphate. Org. Biomol. Chem..

[bib38] McCoy A.J., Gross-Kunstleve R.W., Adams P.D., Winn M.D., Storoni L.C., Read R.J. (2007). Phaser crystallographic software. J. Appl. Crystallogr..

[bib39] Milne J.L., Shi D., Rosenthal P.B., Sunshine J.S., Domingo G.J., Wu X., Brooks B.R., Perham R.N., Henderson R., Subramaniam S. (2002). Molecular architecture and mechanism of an icosahedral pyruvate dehydrogenase complex: a multifunctional catalytic machine. EMBO J..

[bib40] Muller Y.A., Lindqvist Y., Furey W., Schulz G.E., Jordan F., Schneider G. (1993). A thiamin diphosphate binding fold revealed by comparison of the crystal structures of transketolase, pyruvate oxidase and pyruvate decarboxylase. Structure.

[bib41] Murshudov G.N., Vagin A.A., Dodson E.J. (1997). Refinement of macromolecular structures by the maximum-likelihood method. Acta Crystallogr. D Biol. Crystallogr..

[bib42] Nakai T., Nakagawa N., Maoka N., Masui R., Kuraitsu S., Kamiya N. (2004). Ligand-induced conformational changes and a reaction intermediate in branched-chain 2-oxo acid dehydrogenase (E1) from *Thermus thermophilus* HB8, as revealed by X-ray crystallography. J. Mol. Biol..

[bib43] Nemeria N., Arjunan P., Brunskill A., Sheibani F., Wei W., Yan Y., Zhang S., Jordan F., Furey W. (2002). Histidine 407, a phantom residue in the E1 subunit of the *Escherichia coli* pyruvate dehydrogenase complex, activates reductive acetylation of lipoamide on the E2 subunit. An explanation for conservation of active sites between the E1 subunit and transketolase. Biochemistry.

[bib44] Nemeria N., Baykal A., Joseph E., Zhang S., Yan Y., Furey W., Jordan F. (2004). Tetrahedral intermediates in thiamin diphosphate-dependent decarboxylations exist as a 1′,4′-imino tautomeric form of the coenzyme, unlike the michaelis complex or the free coenzyme. Biochemistry.

[bib45] Nemeria N., Korotchkina L., McLeish M.J., Kenyon G.L., Patel M.S., Jordan F. (2007). Elucidation of the chemistry of enzyme-bound thiamine diphosphate prior to substrate binding: defining internal equilibria among tautomeric and ionization states. Biochemistry.

[bib46] Nemeria N., Chakraborty S., Baykal A., Korotchkina L.G., Patel M.S., Jordan F. (2007). The 1′,4′-iminopyrimidine tautomer of thiamin diphosphate is poised for catalysis in asymmetric active centers on enzymes. Proc. Natl. Acad. Sci. USA.

[bib47] Patel M.S., Harris R.A. (1995). Alpha-keto acid dehydrogenase complexes: nutrient control, gene regulation and genetic defects. Overview. J. Nutr..

[bib48] Patel M.S., Harris R.A. (1995). Mammalian alpha-keto acid dehydrogenase complexes: gene regulation and genetic defects. FASEB J..

[bib49] Perham R.N. (1991). Domains, motifs, and linkers in 2-oxo acid dehydrogenase multienzyme complexes: a paradigm in the design of a multifunctional protein. Biochemistry.

[bib50] Perham R.N. (2000). Swinging arms and swinging domains in multifunctional enzymes: catalytic machines for multistep reactions. Annu. Rev. Biochem..

[bib51] Schellenberger A. (1998). Sixty years of thiamin diphosphate biochemistry. Biochim. Biophys. Acta.

[bib52] Schuttelkopf A.W., van Aalten D.M. (2004). PRODRG: a tool for high-throughput crystallography of protein-ligand complexes. Acta Crystallogr. D Biol. Crystallogr..

[bib53] Seifert F., Ciszak E., Korotchkina L., Golbik R., Spinka M., Dominiak P., Sidhu S., Brauer J., Patel M.S., Tittmann K. (2007). Phosphorylation of serine 264 impedes active site accessibility in the E1 component of the human pyruvate dehydrogenase multienzyme complex. Biochemistry.

[bib54] Seifert F., Golbik R., Brauer J., Lilie H., Schroder-Tittmann K., Hinze E., Korotchkina L.G., Patel M.S., Tittmann K. (2006). Direct kinetic evidence for half-of-the-sites reactivity in the E1 component of the human pyruvate dehydrogenase multienzyme complex through alternating sites cofactor activation. Biochemistry.

[bib55] Vakser I.A. (1996). Long-distance potentials: an approach to the multiple-minima problem in ligand-receptor interaction. Protein Eng..

[bib56] Wille G., Meyer D., Steinmetz A., Hinze E., Golbik R., Tittmann K. (2006). The catalytic cycle of a thiamin diphosphate enzyme examined by cryocrystallography. Nat. Chem. Biol..

