# Supplementary material for: Snapshots of Catalysis in the E1 Subunit of the Pyruvate Dehydrogenase Multienzyme Complex
Source: Structure. 2008 Dec 12;16(12):1860–72. doi: 10.1016/j.str.2008.10.009 (PMC2663715; doi:10.1016/j.str.2008.10.009)
Supplement: Document S1. Supplemental Experimental Procedures, Supplemental References, and Three Figures [file mmc1.pdf]

Structure *16*

Supplemental Data

Snapshots of Catalysis in the E1 Subunit

# of the Pyruvate Dehydrogenase Multienzyme Complex

Xue Yuan Pei, Christopher M. Titman, René A.W. Frank, Finian J. Leeper, and Ben F. Luisi

SUPPLEMENTAL EXPERIMENTAL PROCEDURES

Enzymatic analysis of Q81α

Q81α supports the diphosphate group of the ThDP and is not expected to play an important role in the catalytic process. Replacing Q81α with glutamate, corresponding to the exchange of an amide for a carboxylate, was found to increase slightly DCPIP activity. The boost in activity in the DCPIP assay might be caused by the re-organization of solvent in the pocket to increase access for the substrates and DCPIP. The Q81Eα mutant showed a similar level of activity to the wild type in activity of the full PDH complex (Table 3, main text).

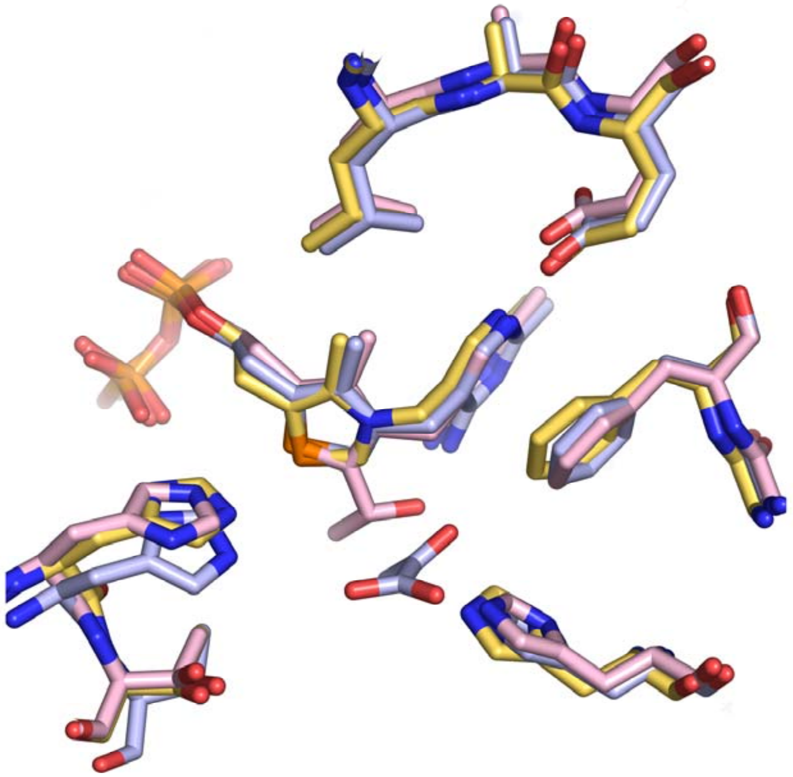

Fig S1

Figure S1. View of the Active Site in the Common Reference Frame of the Cofactor
Shown are the PDH structures with the natural ThDP (carbon atoms in yellow), the pyruvate complex (grey), and the mimic of enamine-ThDP (pink).

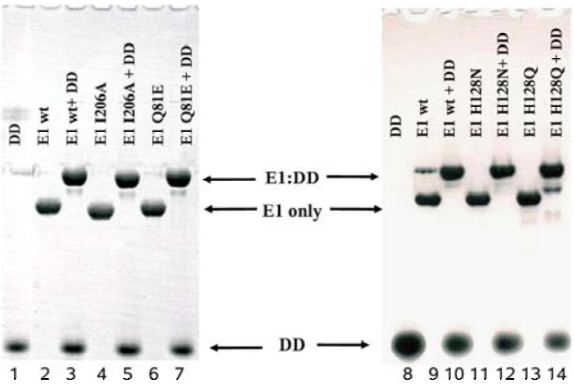

Figure S2. Interactions of the Wild-Type and Mutant E1p Enzymes with the E2p Recognition Domain

The E1p active-site mutants (H128Nβ, H128Qβ, I206Aα, and Q81Eα) were evaluated for their abilities to bind to E2p using electrophoretic mobilities in native gels in the presence and absence of the E2 lipoyl-PSBD di-domain. For all mutants, a band-shift was apparent, showing that the mutant E1p can bind the E2p di-domain, and implying that they are capable of assembling within the PDH multienzyme complex. These results are consistent with an earlier study that showed that active site mutations H128Aβ and H271Aα had no effect on binding of the E2p PSBD in *G. stereothermophilus* E1p (Fries et al., 2003). Interactions were evaluated by mobility shifts using 8% polyacrylamide Tris-glycine buffered gels under native conditions. The di-domain of the PSBD and lipoyl domain was used for these experiments. Binding was observed for wild type and each of the mutants. Lane 1, E2p di-domain alone; lane 2, E1p wild type; lane 3, E1p wild type and E2p di-domain; lane 4, E1p I206Aα mutant alone; lane 5, E1p I206Aα and E2p di-domain; lane 6, E1p Q81Eα mutant; lane 7, E1p Q81Eα mutant and E2p di-domain; lane 8, E2 di-domain; lane 9, E1p wild type; lane 10, E1p wild type and E2p di-domain; lane 11, H128Nβ mutant; lane 12, H128Nβ mutant and E2p di-domain; lane 13, H128Qβ mutant; lane 14, H128Qβ mutant and E2 di-domain.

Fig S3

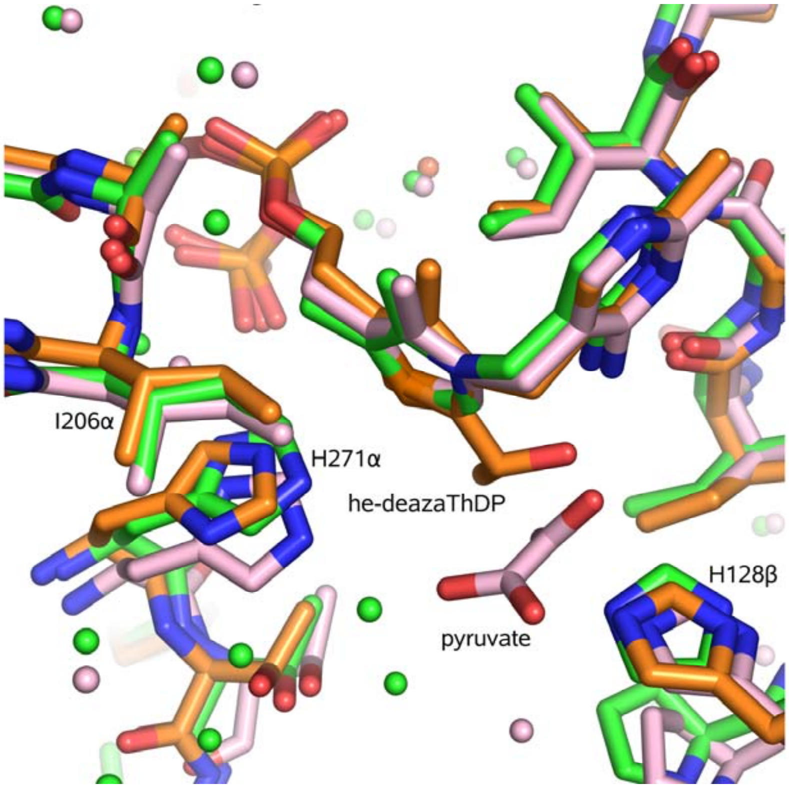

Figure S3. Comparison of the Hydration Pattern at the Active Site
Shown are the pyruvate-bound complex (pink) and the mimic of the enamine-ThDP intermediate (orange), and the wild type structure (green). The overlay shows that the hydration pattern has changed with the different co-factors and ligands (see also discussion in main text).

SUPPLEMENTAL REFERENCE

Fries, M., Jung, H.I., and Perham, R.N. (2003) Reaction mechanism of the heterotetrameric (α2β2) E1 component of 2-oxo acid dehydrogenase multienzyme complexes. Biochemistry *42*, 6996-7002.
